# Homœopathic Medical College of Missouri

**Published:** 1894-07

**Authors:** 


### HOMŒOPATHIC MEDICAL COLLEGE OF MISSOURI.

The Homœopathic Medical College of Missouri held its thirty-fifth annual commencement March 22d at the Pickwick Theatre, St. Louis, Mo. The exercises were attended by a very large assemblage of ladies and gentlemen, many of whom were relatives and friends of the graduates.

The officers of the college are W. A. Edmonds, President; A. H. Schott, Vice-President; L. C. McElwee, Secretary; N. O. Nelson, Charles Cabanne, James B. Case, I. M. Mason, F. W. Brockman, A. B. Howard, E. O. Stanard, and F. G. Niedringhaus, Honorary Board of Trustees; W. C. Richardson, Dean; and L. C. McElwee, Registrar.

The programme was conducted by Dr. Wm. C. Richardson, who introduced Rev. E. B. Chappell, of the Lafayette Park Methodist Church, by whom the invocation was offered. Dr. Richardson, Dean of the college, made the annual report of the institution, which showed that, notwithstanding the general decrease of the number of medical students this year, the enrollment of the Homœopathic College was larger than ever before by fifteen per cent. In all other points the report indicated an equal degree of progress.

Miss Agnes Gray delighted the audience with a few well-rendered violin solos, after which Dr. W. A. Edmonds conferred the degree of Doctor of Medicine on the members of the graduating class in most eloquent form.

A tenor solo, "For All Eternity," was rendered by George H. Kerswill.

The awarding of prizes and distributing of flowers, which was no small task, was conducted by Dr. I. D. Foulon, Professor of Medical Jurisprudence, who rendered this exercise doubly interesting by many well-chosen humorous hits. The Faculty prize, which consisted of Hahnemann's *Materia Medica Pura*, was awarded to Dr. E. J. Hall, who stood highest at the final examination. This gentleman also captured the Gentry prize and the Reid prize, the former consisting of Gentry's *Concordance Repertory*, and the latter a case of high potencies. The obstetrical prize, a pair of Comstock's obstetrical forceps, was awarded to Dr. John M. Lockhead. The gynæcological prize, a Hale's speculum, was won by Dr. Thomas M. Turner, and Luytie's prize, a volume of Bell, to the lady graduate of the highest standing, was won by Mrs. Marguerite G. Squire. Six other individual prizes, from as many prominent physicians and pharmacists, were distributed among the students.

The floral presents were very profuse, each graduate taking away from two to ten bouquets, some of which were very large and handsome in design as well as in composition.

Dr. W. C. Richardson announced that the management of the Children's Hospital, at Jefferson Avenue and Adams Street, had for many years chosen its resident physician from among the graduates of this college, and that the honor on this occasion fell upon Dr. John M. Lockhead.

Rev. Frank G. Tyrrell, of the Central Christian Church, made the address on behalf of the Faculty, and valedictory, and the exercises closed by a violin solo by Miss Agnes Gray. Mr. Charles Kunkel rendered the violin solo, "Old Folks at Home," which was highly appreciated by the audience.

The graduates were Wm. H. Badger, Robert E. Graul, Edward J. Hall, Alfred W. Hayward, Louis W. Minick, John M. Lockhead, George C. Mohler, Scott E. Parsons, Ralph B. Raney, Jesse S. Sargent, Thomas M. Turner, Marguerite G. Squire, Ada Walton, and Mary E. Wolfer.

### ALUMNI ASSOCIATION.

The annual reunion and banquet of the Alumni Association of the Homœopathic Medical College of Missouri was held at the Mercantile Club. A number of distinguished guests from abroad were present. The officers for the ensuing year were elected as follows: Dr. James A. Campbell, President; Dr. C. J. Luyties, First Vice-President; Dr. W. A. Edmonds, Second Vice-President; Dr. W. B. Thompson, Secretary; and Dr. C. A. Carriere, Treasurer. Dr. W. C. Richardson acted as toast-master, and the following toasts were responded to: "Our College," W. A. Edmonds; "Advances in Medicine," A. Merrill; "The Alumni Association," W. John Harris; "The Physician as I Have Known Him," Rev. John Snyder; "Surgery and Homœopathy," W. B. Morgan; "Homœopathic Literature," Irenæus D. Foulon; "Student Days," A. H. Schott; "The New M. D.," J. S. Sargent.

